# Supplementary material for: Ecosystem antifragility: beyond integrity and resilience
Source: PeerJ. 2020 Feb 11;8:e8533. doi: 10.7717/peerj.8533 (PMC7020813; doi:10.7717/peerj.8533)
Supplement: Supplemental Information 1 [file peerj-08-8533-s001.pdf]

Supplementary Materials: Ecosystem Antifragility: Beyond Integrity and Resilience.

M. Equihua[2,*], M. Espinosa[8,*], C.Gershenson[3,5,6,*], O. López-Corona[1,2,3,*], M. Munguía[7,*], O. Pérez-Maqueo[2,*], and E. Ramírez-Carrillo[4,*]

[1]**Cátedras CONACyT, Comisión Nacional para el Conocimiento y Uso de la Biodiversidad (CONABIO), CDMX, México**
[2]**Red ambiente y sostenibilidad, Instituto de Ecología A.C., Xalapa, México**
[3]**Centro de Ciencias de la Complejidad (C3), Universidad Nacional Autónoma de México, CDMX, México**
[4]**Facultad de Psicología, Universidad Nacional Autónoma de México, CDMX, México**
[5]**IIMAS, Universidad Nacional Autónoma de México, CDMX, México**
[6]**ITMO University, St. Petersburg, 199034, Russian Federation.**
[7]**Comisión Nacional para el Conocimiento y Uso de la Biodiversidad (CONABIO), CDMX, México**
[8]**Doctorado en Ciencias Sociales y Humanidades, UAM-Cuajimalpa.**

Corresponding author:
Following the Hardy-Littleton rule, all authors will appear in alphabetical order. And all are corresponding authors*

Email address: equihuam@gmail.com; mariana.espinosa.aldama@gmail.com; cgg@unam.mx; olopez@conacyt.mx; mariana.munguia@conabio.gob.mx; octavio.maqueo@gmail.com; elviarc@gmail.com

ABSTRACT

We review the concept of ecosystem resilience in its relation to ecosystem integrity from an information theory approach. We summarize the literature on the subject identifying three main narratives: ecosystem properties that enable them to be more resilient; ecosystem response to perturbations; and complexity. We also include original ideas with theoretical and quantitative developments with application examples. The main contribution is a new way to rethink resilience, that is mathematically formal and easy to evaluate heuristically in real-world applications: ecosystem antifragility. An ecosystem is antifragile if it benefits from environmental variability. Antifragility therefore goes beyond robustness or resilience because while resilient/robust systems are merely perturbation-resistant, antifragile structures not only withstand stress but also benefit from it.

1 SURVEY METHODOLOGY

It has recently been shown that Web of Science and Scopus is invisible to a big proportion of highly-cited papers in the social sciences and humanities. And even when the percentage of missing highly cited papers in Web of Science (WOS) and Scopus is in the natural, lives and social sciences. The Spearman quotation correlation coefficients in Google Scholar are more powerful in all fields compared to Web of Science and Scopus. The researchers conclude that highly cited papers available in the inclusive Google Scholar database actually show important deficiencies in the coverage of the Web of Science and Scopus in certain study fields. Consequently, using these selective databases to calculate bibliometric indices based on the number of highly cited papers could generate partial evaluations in poorly covered fields Martín-Martín et al. (2018).

For these reasons we choose Google Scholar as the search engine in which we use the term: "Ecosystem Resilience" *AND* "Information theory" *AND* " "Ecosystem Integrity". After excluding patents and citations we end up with 20 items. Of those 20 co-occurrences, books and patents were discarded, leaving

10 entries that where fully read. Finally, only 7 items were selected to be analyzed and included in the present review. However, Google Scholar, does not provide easy access to cited references, which indicate the knowledge background of the selected items. For that reason, the analysis was completed with data from the Web of Science and the Astrophysics Data System to visualise their cited references network using Science of Science (Sci2) Tool and Gephi. To further analyze the literature set, a text corpus was assembled taking the text of the title and abstract of target documents as a bag of words. This small corpus was then processed with latent Dirichlet allocation (LDA) and latent semantic analysis (LSA) techniques.

FINDINGS

Basic scientometrics i.e word clouds

In Table 1 we show basic metrics for papers selected and in Fig. 1 the results of applying LDA analysis to this corpus, based on Sievert and Shirley (2014) with an interactive viz that can be opened in any browser. A video explaining the use of this kind of viz could be found in here. According with LDA, papers can be allocated to the topics as indicated in Table 1; although, topics can be concurrently present in any paper.

Source	Google Scholar
Papers	7
Citations	454
Years	23
Cites_Year	19.74
Cites_Paper	64.86
Cites_Author	149.78
Papers_Author	1.95
Authors_Paper	4
h_index	7
g_index	7
hc_index	6
hI_index	1.75
hI_norm	6
AWCR	71.34
AW_index	8.45
AWCRpA	17.93
e_index	20.12
hm_index	1.95
QueryDate	2019-02-04
Cites_Author_Year	6.51
hI_annual	0.26
h_coverage	100
g_coverage	100
star_count	3
year_first	1996
year_last	2018
ECC	454

Table 1. Table shows basic metrics for the Google Scholar search.

In Fig. 2 we present a main axes plot based in a latent semantic analysis (LSA). Both LDA and LSA suggest it is possible to recognize four groups in the corpus, which are further discussed in the section analyzing perceived literature narratives below. In addition, the analysis of the citation network reveals several unconnected sub-groups, while the cited references in general are very poorly connected. We interpret this findings as evidence of poor interdisciplinary crossover on the conceptual development of ecological resilience and integrity, which prompted our interest in the issues we discuss in this paper.

In Fig.4 we show a TreeMap for the type of documents that cite the set of reviewed ones; in Fig5 The organizations of origin for the documents that cite the set of reviewed ones; in Fig.6 we show the number

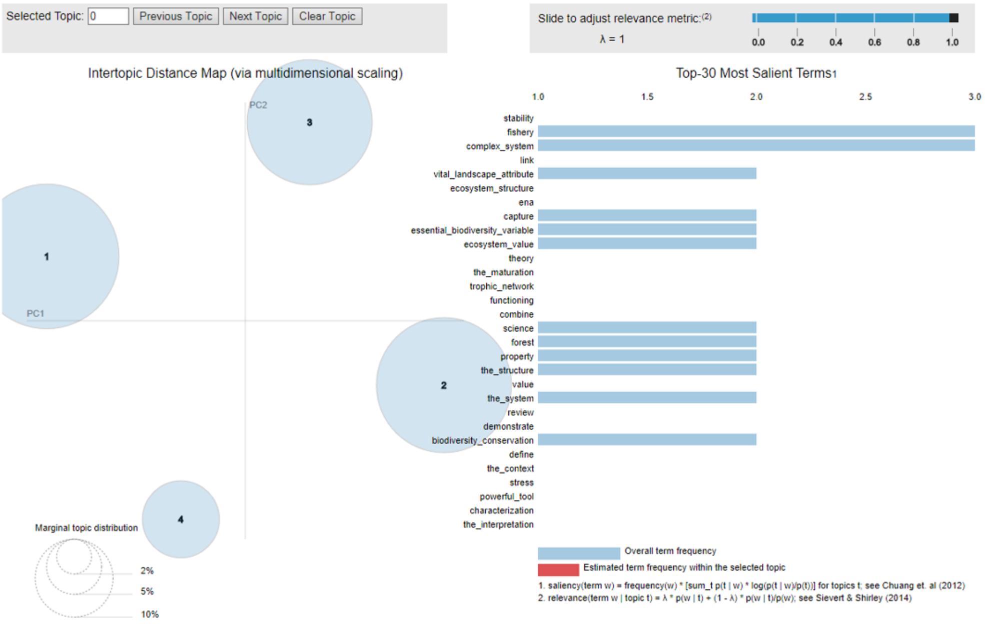

Figure 1. Latent Dirichlet Allocation (LDA) analysis based on Sievert and Shirley (2014) with an interactive viz to be opened in any browser. In short, the interface has two main panels. Topic pattern on the left and terms frequencies on the right. The left panel shows a general perspective of the discovered subjects indicating how common each is in the corpus (the set of papers) and how they relate to each other ; the subjects are plotted as circles whose centers are characterized by the computed range between the subjects (projected into 2 dimensions). The prevalence in the corpus of each topic is proportional to the circle size. The right panel has a bar chart showing the meaning of terms, informative of the possibly interpretation of the topics essence. You can pick any subject interactively and find out the function of terms in it. Two overlaid bars are shown at each place when pointing to a subject, displaying the topic-specific frequency of each word (in red) and the corpus-wide frequency (in blue gray). When no topic is selected, the right panel displays the top 30 most salient terms for the dataset. A video explaining the use of this kind of viz could be found in here

of documents that cite the set of reviewed ones; and in Fig.7 the distribution of documents that cite the set of reviewed ones in terms of research field.

REFERENCES

Martín-Martín, A., Orduna-Malea, E., and López-Cózar, E. D. (2018). Coverage of highly-cited documents in google scholar, web of science, and scopus: a multidisciplinary comparison. *arXiv preprint arXiv:1804.09479*.

Sievert, C. and Shirley, K. (2014). Ldavis: A method for visualizing and interpreting topics. In *Proceedings of the workshop on interactive language learning, visualization, and interfaces*, pages 63–70.

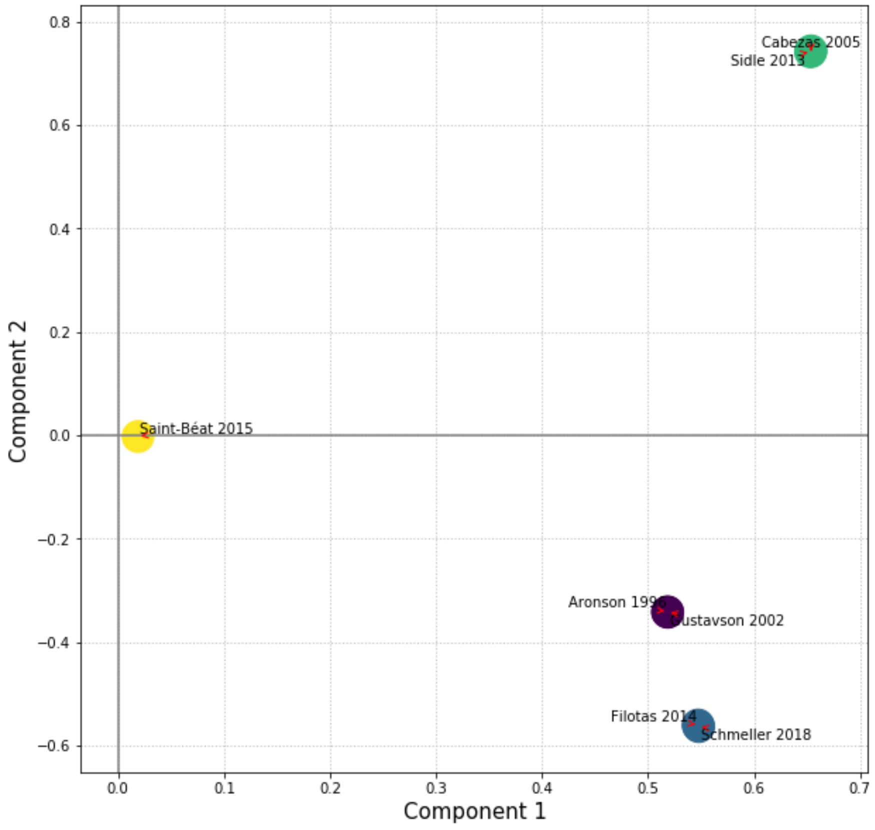

Figure 2. Latent semantic analysis of papers

LDA Topic	Paper
1	Cabezas 2005
1	Sidle 2013
2	Aronson 1996
2	Gustavson 2002
3	Saint-Béat 2015
4	Filotas 2014
4	Schmeller 2018

Table 2. Allocation of paper to dominant topic found by LDA.

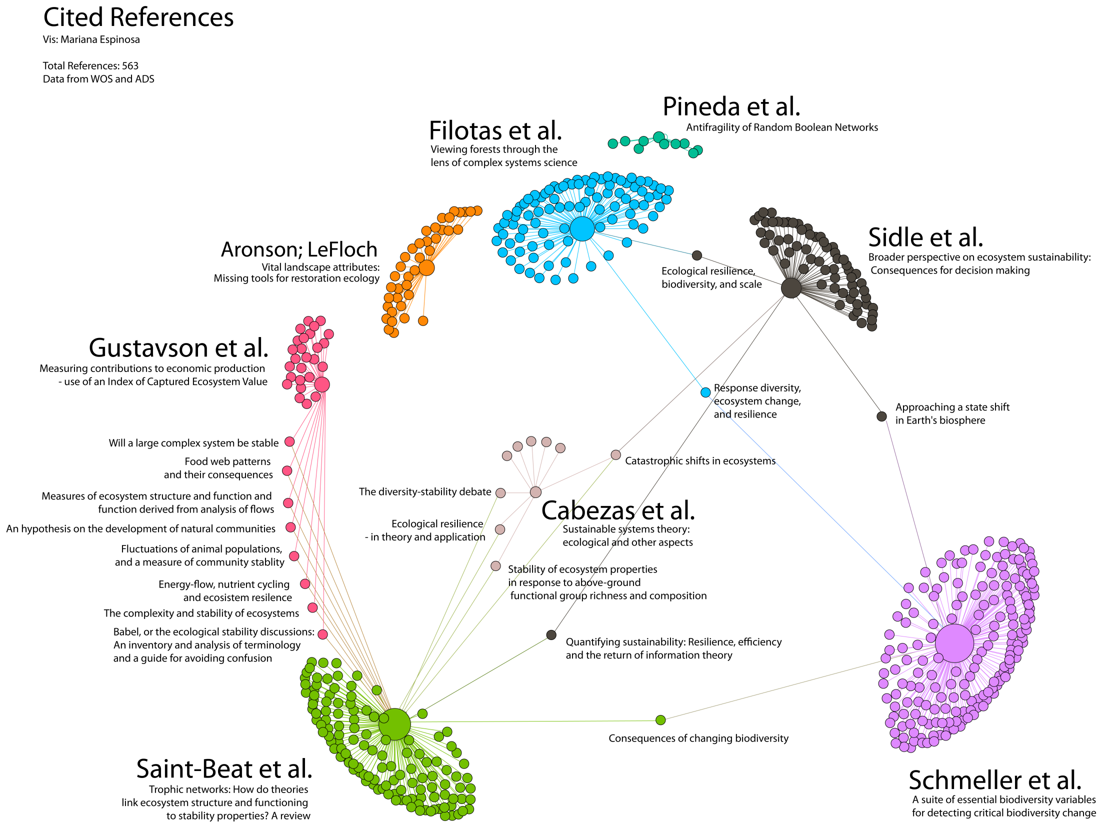

Figure 3. The citation network for the selected paper including them

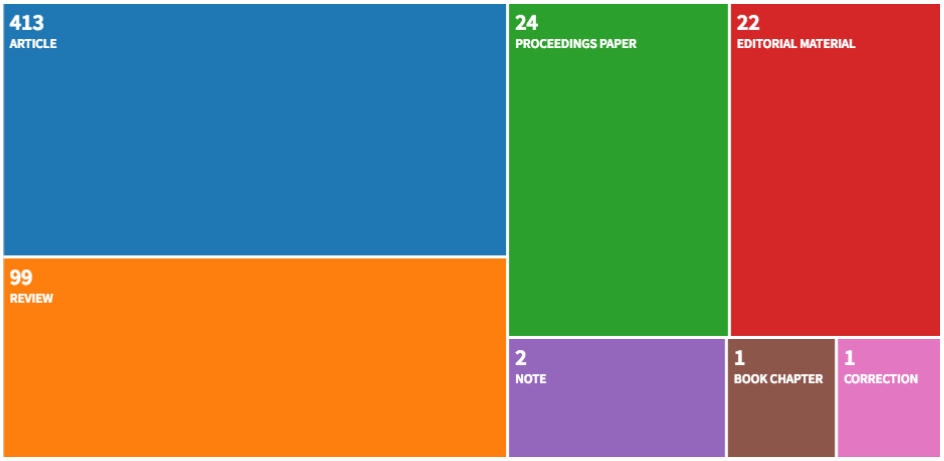

Figure 4. TreeMap for the type of documents that cite the set of reviewed ones

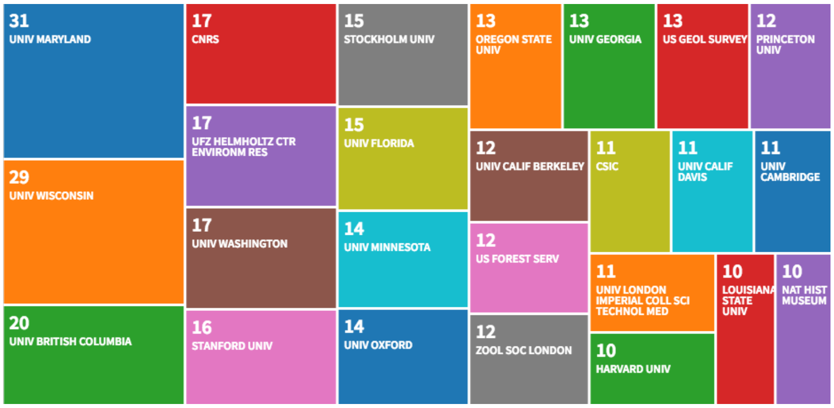

Figure 5. Organizations of origin for the documents that cite the set of reviewed ones

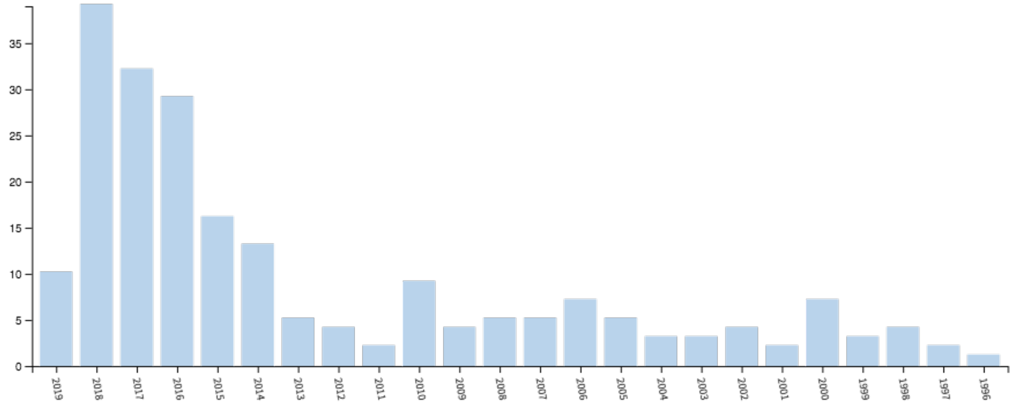

Figure 6. Number of documents that cite the set of reviewed ones

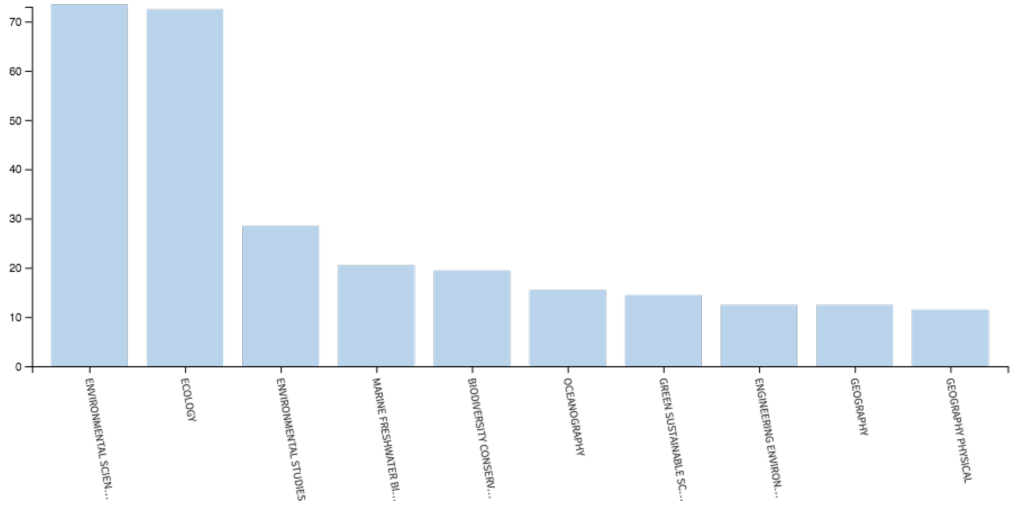

Figure 7. Distribution of documents that cite the set of reviewed ones in terms of research field
